# An Introduction to Machine Learning Approaches for Biomedical Research

**DOI:** 10.3389/fmed.2021.771607

**Published:** 2021-12-16

**Authors:** Juan Jovel, Russell Greiner

**Affiliations:** ^1^The Metabolomics Innovation Centre, University of Alberta, Edmonton, AB, Canada; ^2^Faculty of Science-Computing Science, University of Alberta, Edmonton, AB, Canada

**Keywords:** machine learning, biomedical research, supervised learning, unsupervised learning, reinforcement learning

## Abstract

Machine learning (ML) approaches are a collection of algorithms that attempt to extract patterns from data and to associate such patterns with discrete classes of samples in the data—e.g., given a series of features describing persons, a ML model predicts whether a person is diseased or healthy, or given features of animals, it predicts weather an animal is treated or control, or whether molecules have the potential to interact or not, etc. ML approaches can also find such patterns in an agnostic manner, i.e., without having information about the classes. Respectively, those methods are referred to as supervised and unsupervised ML. A third type of ML is reinforcement learning, which attempts to find a sequence of actions that contribute to achieving a specific goal. All of these methods are becoming increasingly popular in biomedical research in quite diverse areas including drug design, stratification of patients, medical images analysis, molecular interactions, prediction of therapy outcomes and many more. We describe several supervised and unsupervised ML techniques, and illustrate a series of prototypical examples using state-of-the-art computational approaches. Given the complexity of reinforcement learning, it is not discussed in detail here, instead, interested readers are referred to excellent reviews on that topic. We focus on concepts rather than procedures, as our goal is to attract the attention of researchers in biomedicine toward the plethora of powerful ML methods and their potential to leverage basic and applied research programs.

## Introduction

**Figure 1:**
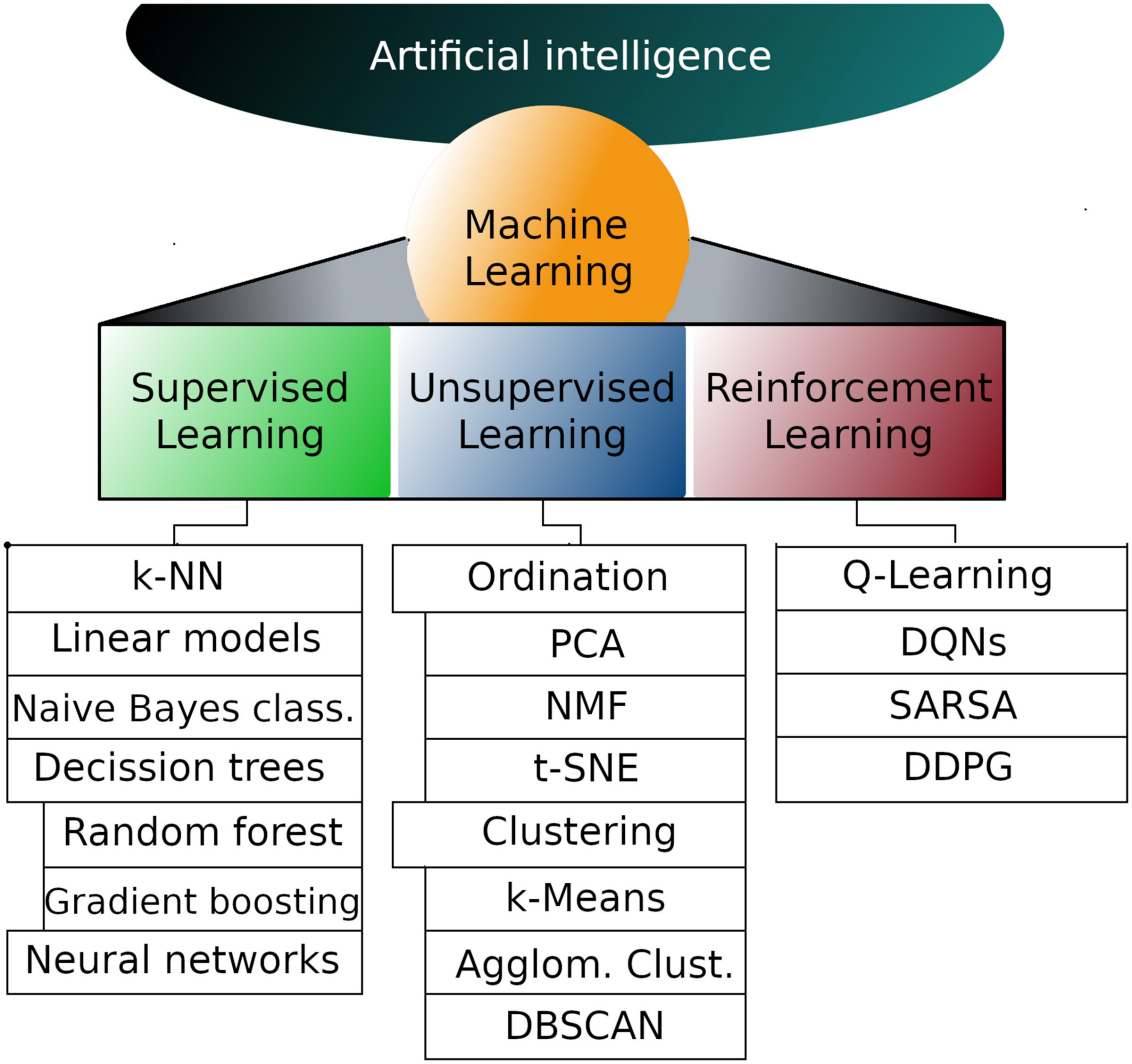
Representative machine learning algorithms. Machine learning is a subfield of artificial intelligence and can be divided into supervised, unsupervised and reinforcement learning. The list of algorithms in each subfield is not exhaustive but instead are the most popular algorithms in each subfield. k-NN, k nearest neighbors; PCA, Principal components analysis; NMF, Non-negative matrix factorization; t-SNE, T-distributed stochastic neighbor embedding; DQNs,Deep Q networks; SARSA, State-action-reward-state-action; DDPG, Deep deterministic policy gradient.

Machine learning (ML) is a branch of artificial intelligence (AI) that deals with the implementation of computational algorithms that improve performance upon experience; in other words, a ML system learns from data (1, 2). In its classical definition, ML approaches include three types of knowledge acquisition: supervised learning, unsupervised learning and reinforcement learning (3) (Figure 1).

In supervised learning, an algorithm trains a statistical model, which in turn is able to make predictions about an unlabeled instance. During training, a column of data containing the answer (label or target) is used to supervise the learning process (4, 5). For example, given a data set of cancer patients, the label column could contain tumor classes indicating whether the tumor of a patient ended up being benign or malignant. Alternatively, the label column could indicate the number of people affected by an infectious disease in each country of the world. In both cases, the model learns to associate the values of a series of predictor variables, known as “features” in the ML jargon, with the label variable. Once trained, the model can predict the label value in new data only from the values of features. It is, a model would be able to predict whether an unclassified tumor will turn benign or malignant, or the number of infected people in each country at a future time point.

Unsupervised learning, as suggested by its name, does not rely on labels, but purely on values of features, i.e., the intrinsic structure of data (5, 6). The emphasis here is on the extraction of knowledge through a process of pattern discovery. An obvious advantage of unsupervised learning, compared to its supervised counterpart, is that the onerous and often expensive task of creating accurate labels is omitted, and so are the potential biases introduced thereby. A drawback of unsupervised learning is that it is difficult to assess the quality of what the model has learned, because it is not known what the correct answer should be (7). In many scenarios, unsupervised learning can be applied to assign labels to multidimensional data, and subsequently supervised learning is applied on the resulting dataset, to gain additional insight. A typical application of unsupervised learning is the ordination of transcriptomes sequenced from single cells to define clusters of cells transcriptionally similar (8). Here, features values correspond to abundance of thousands of transcripts (or genes), samples correspond to cells and there are no labels.

Reinforcement learning (RL) is fundamentally different from the two former approaches because it does not need human-generated data for training, but instead it learns from a trial and error process (9, 10). The algorithm receives feedback from the analytical process itself in the form of rewards when an action contributes to reach the goal, or conversely in the form of penalties when the action does not contribute to reach the proposed goal. The agent (algorithm) aims at maximizing reward and minimizing penalty (11). Implementation of RL is relatively more challenging than supervised and unsupervised learning and for this reason it will not be discussed further here. Readers interested in fundamentals of RL, or in applications of RL to biomedical research, are encouraged to read the following (9–16).

ML approaches have been used for decades, and its conception dates back centuries (17). In recent years, three main technological advances have given renewed impetus to ML (2). First, a substantial increase in computing power, which has been democratized through cloud computing. This has enabled an effervescence of research in ML by developers and theoreticians around the world. Second, high-throughput technologies for data acquisition, including sensors, cameras, DNA and protein sequencing instruments, high-throughput metabolomics, etc., have allowed the accumulation of large amounts of data, which is invaluable for the efficient training of ML models. Third, the emergence of big software companies, including Google, Facebook, Amazon, Microsoft, but also academic institutions, have contributed the most popular ML frameworks (libraries) to the community, including TensorFlow, Keras, PyTorch, Scikit-Learn, Caffe, CNTK, Lasagne, and Theano, among others. Moreover, cloud computing providers like Google, Amazon, Microsoft and IBM, now enable users with Artificial Intelligence algorithms as a Service (AIaaS). Recently, many ML implementations have already contributed to solving real life problems in ways that were not possible before.

In the next section, supervised and unsupervised ML are discussed in more detail and illustrated with examples that facilitate their comprehension. The algorithms developed for each approach are plentiful, and it would be impractical to describe all of them. Instead a handful of the most popular ones are mentioned here in a rather succinct manner and the reader is directed to relevant references for a more comprehensive landscape. This article is not intended for experts in bioinformatics or statistics, but for researchers in life sciences that might be interested in incorporating ML approaches to their research programs. No prior knowledge in computer sciences is required. We hope that this lay introduction will help readers to become conversant with ML fundamentals to ultimately facilitate its adoption.

## Machine Learning Approaches Demystified

### Supervised Learning

Supervised learning deals with two families of problems: classification and regression (5, 7). In the first case, predictions are made about a categorical variable (e.g., cancer type, survival of patients, etc.); in the second case, the label is a numerical variable (e.g., number of infected people, price of a treatment, etc.). Also, a numerical variable can be converted into classes and then handled as a classification problem. Initially, data is divided into two subsets: a larger subset, often 60–75%, which is used for training the model, and a test subset used for prediction and evaluation. Often, separate evaluation and test datasets are used. In those cases, the evaluation dataset is used for tuning the parameters of the model and for feature selection, while the test data set is used for an unbiased evaluation. For simplicity, here we will refer to them just as the test dataset. During training, the labels are shown to the model, so that it learns to associate patterns in the data with specific values of the target column. Once trained, the model can be evaluated on the test data subset, and its performance is determined based on metrics. When a model predicts values of the target variable with high accuracy, it is said that the model generalizes well. The description of some popular supervised learning algorithms follows. Since most algorithms are available for classification and regression analyses, unless otherwise mentioned, that should be assumed to be the case. Currently, supervised learning is by far the most important approach for biomedical research and for this reason it receives special attention here.

#### K-Nearest Neighbors

The k-NN algorithm assumes that similar things are in close proximity to each other. Based on Euclidean distances among samples (or other more sophisticated distance metrics), the k-NN algorithm iteratively assigns samples to clusters, so that samples with similar feature values will be separated by shorter distances and therefore will be assigned to the same cluster (1, 18). However, it is possible that samples clustered together belong to different classes, e.g., samples from benign and malignant tumors might integrate the same cluster; in those cases, newly added samples are assigned to the majority class, through a voting process (e.g., if most samples in that cluster belong to the malignant cancer class, any benign sample erroneously assigned to that cluster, will also be classified as malignant). As proximal samples are added to a cluster with the nearest centroid, centroids are recalculated. The final goal is to minimize inertia, which is the sum of distances between the centroid and the samples inside a cluster. Because the end data structure of a k-NN process is a distance matrix, it is not possible to assess the individual contribution of each feature to the classification process (19).

**Figure 2:**
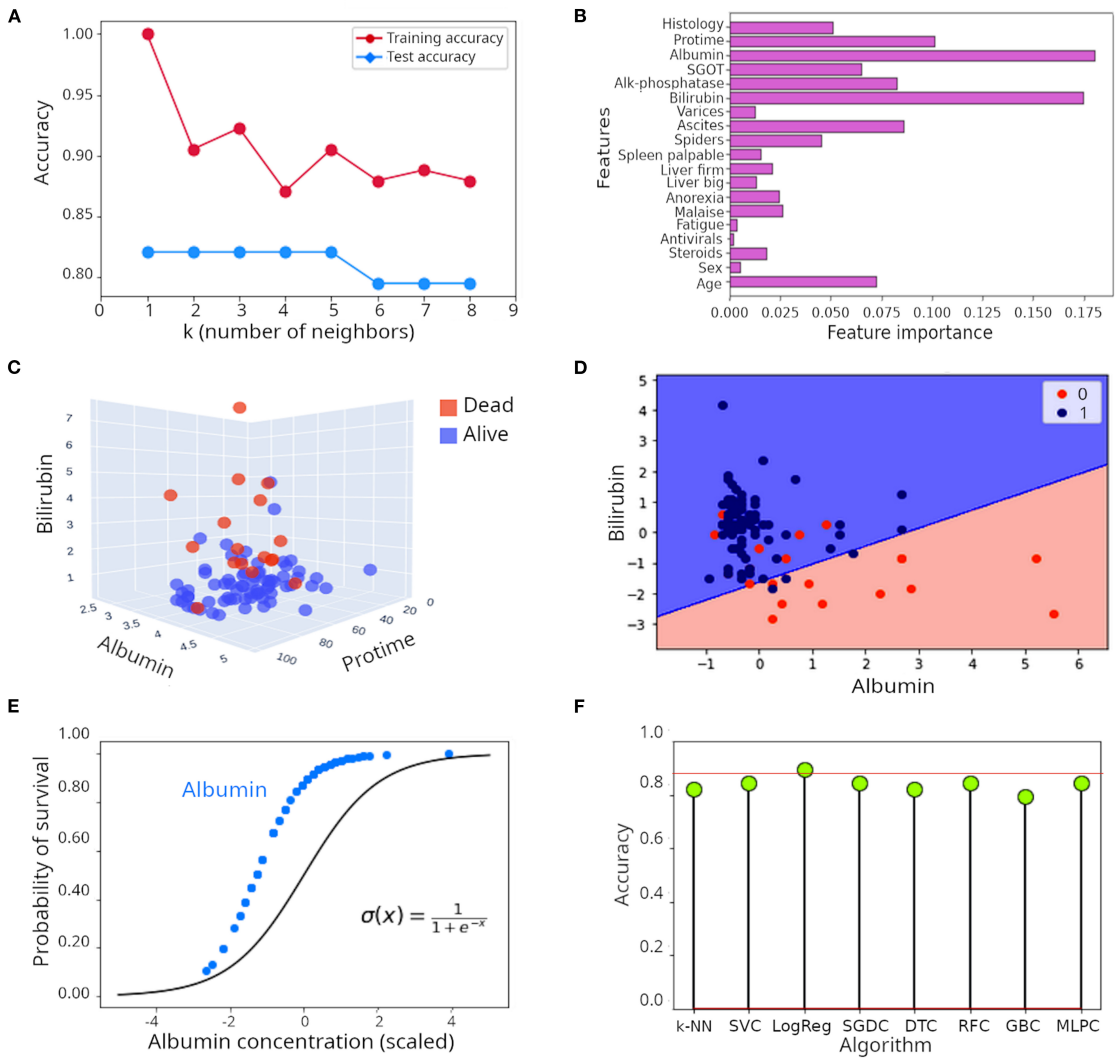
Illustration of supervised learning algorithms. **(A)** Relationship between number of neighbors (k) and accuracy in the k-NN algorithm when applied to the hepatitis dataset. **(B)** Feature importance when the random forest algorithm was applied to the hepatitis dataset. **(C)** Tri-dimensional scatter plot of values of albumin, bilirubin and protime in patients included in the hepatitis dataset. **(D)** Decision surface of the logistic regression model applied to the hepatitis dataset illustrated in a two dimensional plot including only albumin and bilirubin. **(E)** Comparison of the theoretical probability distribution of a logistic regression model with the probability distribution of survival of patients in the hepatitis dataset when only albumin is considered as regressor. **(F)** Lollipop plot of accuracy achieved during classification of survival in the hepatitis dataset. k-NN, k nearest network; SVC, Support vector classifier; LogReg, Logistic regression (R squared); SGDC, Stochastic gradient descent classifier; DTC, Decision tree classifier; RFC, Random forest classifier; GBC, Gradient boosting classifier; MLPC, Multilayer perceptron classifier.

Let's illustrate the k-NN algorithm with a real example. The hepatitis data set (illustrated in Supplementary Figure 1), contains records of 155 patients affected by hepatitis with measurements of a series of clinical variables aimed at helping clinicians with monitoring disease progression. Despite its small size, this is a popular dataset in ML forums because its structure is well-suited for explanation of ML concepts (20, 21). Of those, 32 patients died and 123 survived. Survival is the label here. The optimal number of neighbors (k) to consider in a k-NN model is determined empirically using the training dataset. As seen in Figure 2A, 1–5 neighbors lead to the highest accuracy (82%). Predicting survival of patients correctly 82% of the time is a very encouraging result, but let's see whether that can be improved.

#### Decision Tree-Based Approaches

Using the analogy of a tree to describe this algorithm is very convenient. Let's assume that the whole population of samples corresponds to the root of the tree. The interest then is to split such a population into branches and leaves so that after each split, the subset of samples remaining is more homogeneous than its precursor. More formally, the process consists in splitting precursor populations into decision nodes, which represent attributes of the dataset under analysis (7, 22). A simplistic example involves the classification of birds and mammals from a mixed root population including penguins, eagles, whales and wolves. In the first decision node, we could ask “does it have feathers?” This will separate mammals from birds. In the mammals branch, we could create a second decision node by asking “does it live in the sea?” This will separate whales from wolves. Similarly, in the birds' branch we could ask “does it fly?” This will separate penguins from eagles. That is the basic idea.

The choice of attributes as decision nodes is informed by statistics. Although there are many decision tree algorithms, all will calculate statistics on each attribute and will select the one that best purifies the resulting subsets of samples with respect to the target variable (23–25). Let's use the iterative dichotomiser 3 (ID3) algorithm to illustrate the process (26, 27). The process starts by assigning all samples to the root of the tree. At each iteration, the algorithm calculates entropy (H) and information gain (IG) for each attribute that can be used as a decision node. Here, the entropy of a set is a measurement of randomness of the labels of those instances; the larger the entropy the less homogeneous the subset of samples comprising a node. Conversely, information gain measures how well a given attribute allows separation of training samples according to the target variable. Therefore, the algorithm aims at maximizing and minimizing IG and H, respectively.

When a decision tree algorithm was applied to the hepatitis dataset, an accuracy of 82.1% was obtained, which is quite similar to the results obtained with the k-NN algorithm above. The decision tree obtained is presented in Supplementary Figure 2A. As seen, it placed albumin in the first node (root) and asked whether the content of each sample is smaller or equal to 1.598 and then split samples accordingly. Overfitting occurs when the model learns the training data too well and consequently does not perform well on test data. Intrinsically, decision trees are prone to overfitting, and that reduces their ability to generalize. There are at least two ways to reduce overfitting in decision trees, one is by pruning the tree, i.e., reducing its depth. The other way is to use ensembles of trees, which are implemented in different algorithms like random forest or gradient boosting classifiers (1, 3).

Random forest is a collection of decision trees (7, 19). The assumption is that individual trees will overfit the data in different ways and averaging the results of many trees will reduce overfitting and consequently will improve accuracy of classification. Randomness is injected in two ways. First, the algorithm bootstraps to extract *n* samples with replacement. It means that each data set extracted in this way will be the same size of the original dataset but some samples will be missing while others will be repeated. Second, at each decision node, the algorithm randomly selects a subset of these samples and selects the feature that best splits these samples (3). Gradient boosting works in a similar way, but for the sake of space will not be discussed here. Instead, we encourage reading (28–30). When a random forest algorithm with 60 individual trees was applied to the hepatitis dataset, the accuracy in classification was 85%, which represents a substantial improvement compared to the individual decision tree described above, or to the k-NN approach (accuracy ~ 82.1%). As shown in Supplementary Figure 2B (only considering bilirubin and albumin as predictors), the decision boundary defined by the random forest algorithm is smoother than the one of the single decision tree. A convenient attribute of decision trees, random forest and gradient boosting algorithms is that the individual contribution of each feature to the model can be visualized. In Figure 2B, the individual contribution of each feature to the classification process is presented. Albumin and bilirubin are dominant, followed by protime (which is the time that takes for the blood of a patient to clot in a prothrombin time test), ascites (build up of fluid in the peritoneal cavity) and age. Indeed, when plotting samples in a 3D space including only albumin, bilirubin and protime, a good separation of patients that died from those that survived is achieved (Figure 2C). Although it appears obvious that the content of bilirubin and albumin is higher and lower, respectively, in patients that died, separation of patients based on only these three features is not perfect, which suggests that other features also contribute to the outcome of the disease. Can we still improve on the great performance exhibited by random forest?

#### Linear Models

Linear models are among the simplest, and therefore most popular, models in ML (1, 31). Essentially, a linear model represents the weighted sum of the input features plus an intercept or bias term (32). Given their simplicity, they are ideal for more formally explaining the concept of model or hypothesis. Let's consider the following equation:

**Equation 1**. General equation of linear models for regression.


$$\begin{array}{l} ŷ=w{\left[0\right]}^{*}x\left[0\right]+w{\left[1\right]}^{*}x\left[1\right]\ldots +w{\left[n\right]}^{*}x\left[n\right]+b=b+\overset{N}{\underset{i=1}{\sum }}w_{i}x_{ij} \end{array}$$

We see that each of the features (X) is weighted in the sum by a value *w* and the whole line has a bias equal to *b*. It means that the contribution of each feature to the model may be different. Generally, there will be a difference between the predicted target values and the real ones, in other words a cost associated with the process of mapping. The magnitude of such cost can be estimated by a cost function, using an estimator like the mean squared error (MSE). The problem becomes one of finding the parameters in the model that minimize the costs. This can be achieved using another function called gradient descent (33, 34).

In a two-dimensional space, the equation above defines a line which starts at *b* and extends upwards or downwards depending on the slope (*w*). In three dimensions, the output of the function is a plane, and in multidimensional spaces is a hyperplane. The problem is that the relationship between features and target is often non-linear, and therefore linear models have reduced predictive potential to explain such relationships. Most linear models are used for regression analysis and therefore are not suitable to predict survival in our hepatitis example. There are also linear models for classification of categorical target variables, logistic regression (35) and linear support vector machines (SVM) being the most popular of them. We will discuss classification with logistic regression here, for a discussion on SVM, the reader is encouraged to review (7, 19).

Logistic regression is very popular in biomedical research (36), and is often used to predict whether a set of conditions will result, or not, in disease or death of patients. Logistic regression is a non-linear function that models the probability of belonging to a class or another based on a linear combination of features (36). For a target variable with two outcomes, as in our hepatitis example (death or alive), the logistic regression equation is as follows:

**Equation 2**. Logistic regression equation for binary target variables.


$$\begin{array}{l} ŷ=\frac{e^{X}}{1+e^{X}} \end{array}$$

where *x* corresponds to the linear regression equation presented above. The linear regression equation defined by the exponent *x* gives rise to the *logit* or logarithm of the odds:

**Equation 3**. Log odds in logistic regression.


$$\begin{array}{l} \ln\left(\frac{ŷ}{1-ŷ}\right)=b+\overset{N}{\underset{i=1}{\sum }}w_{i}x_{ij} \end{array}$$

In other words, the linear model defines the natural logarithm of the probability of being in a class divided by the probability of being in the other class. When we applied logistic regression to the hepatitis dataset, an *R*^2^ of 0.90 (90%) was obtained, which is a pretty satisfactory result. To illustrate its intrinsic linear nature, we plotted the decision surface of this model only for albumin and bilirubin (Figure 2D). The probability distribution of survival based only on albumin, which is the most influential feature, closely mirrored the theoretical probability distribution of logistic regression (Figure 2E). As can be seen, there are no values for albumin concentration below some point, likely because that is the threshold of lethality (Figure 2E). It is important to note that the R squared statistic is not directly comparable to the accuracy of a model; it does not reflect the prediction power of the model, instead, it represents the proportion of the variance explained by the model.

#### Deep Learning

The approaches discussed so far belong to the classical ML realm. Artificial neural networks (ANN; often referred to as feed-forward neural nets) owe their name to the fact that they aspire to emulate the interconnected system of neurons. ANNs are central to deep learning (37). Although ANNs were initially proposed more than 70 years ago (38), interest in them has recently been revived mainly due to the exponential increase seen in computer power and data for training ANNs. This led to successful implementations that further boost their relevance.

**Figure 3:**
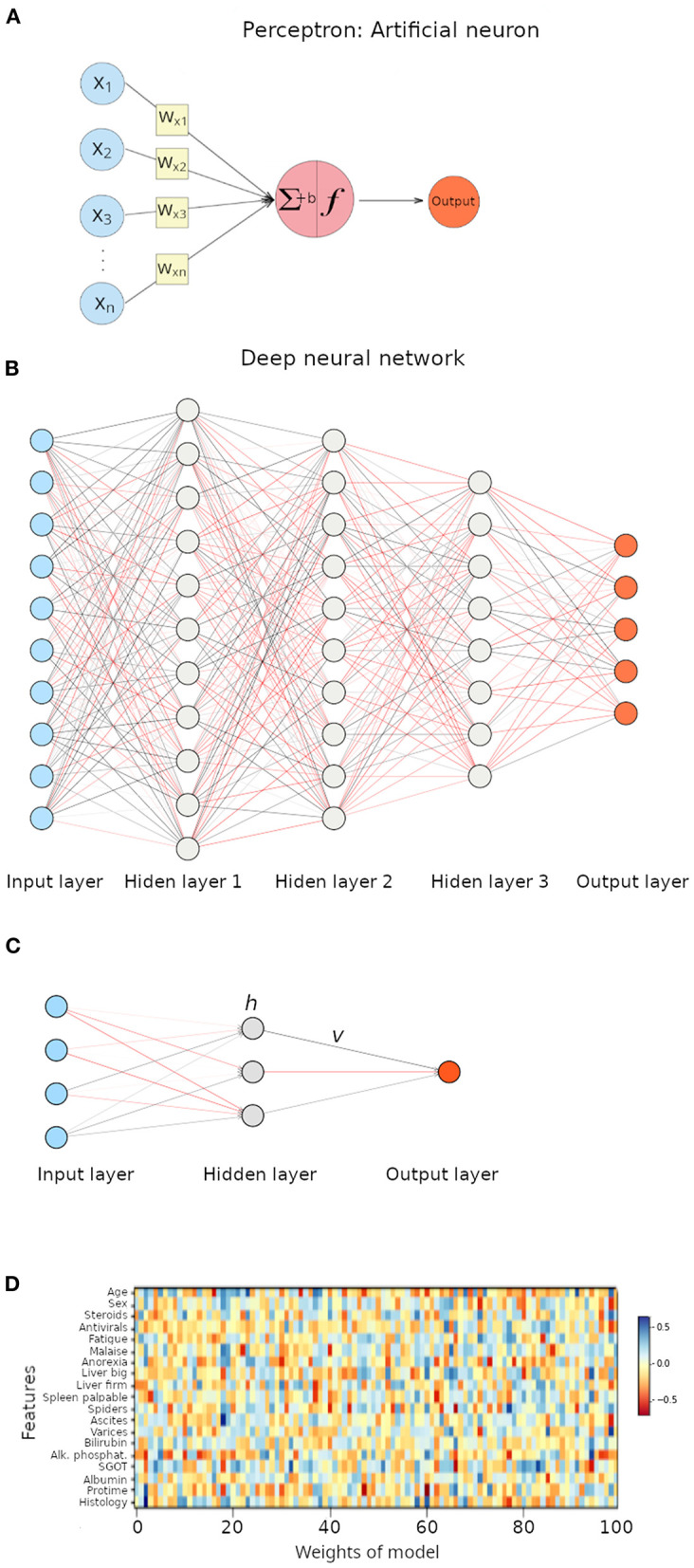
Illustration of artificial neural networks (ANNs). **(A)** Perceptron, a neural network with a single neuron. ∑ represents the weighted sum, *f* represent the activation function, and *b* represents the bias term. **(B)** Deep neural network with three hidden layers. Each interconnected node represents a neuron. **(C)** Neural network with a single hidden layer. **(D)** Heat map representation of weights in the multilayer perceptron model applied to the hepatitis dataset.

The perceptron is an ANN with a single neuron (Figure 3A), and it is an ideal architecture to explain the foundations of neural networks (39). As seen in Figure 3A, the perceptron receives numeric inputs and calculates a weighted sum (as explained above for linear models of regression). It means that each input value is multiplied by a weight and then all are summed, along with a bias, to finally produce an output. In other words, the weights estimate how large a change in the output is expected to be when the input changes (i.e., the relative contribution of each feature to the output), while the bias allows shifting the activation function by a constant to better fit the model to the data. The reader probably already noticed that such a description is simply a linear model of regression. ANNs differ from classical linear models in what is called an activation function (Figure 3A), and weights are calculated in a different manner. As biological neurons, an artificial neuron has to decide, using the activation function, whether it gets activated or not, based on the magnitude of the stimulus received. Thus, the equation of a perceptron is the linear regression equation multiplied by an activation function.

**Equation 4**. Equation of a perceptron output.


$$\begin{array}{l} ŷ=f\left(b+\overset{N}{\underset{i=1}{\sum }}w_{i}x_{ij}\right) \end{array}$$

The activation function can be as simple as the implementation of a threshold (i.e., step function) that acts like a switch that turns the neuron on and off if a threshold value is exceeded, or there can be other non-linear transformations that allow ANNs to learn complex data patterns. The true complexity is based in many layers applying simple functions. Non-linear transformations include the rectified linear function (ReLu; which despite its name is non-linear), the sigmoid function (the same as the logistic regression) and hyperbolic tangent function (Tanh), among others (3, 40, 41).

Deep learning relies on deep neural networks (1, 19). Unlike perceptrons, a deep neural network contains many neurons, each of them connected to neurons in other layers by edges that represent the weights and each neuron has an activation function (Figure 3B). The weighted sum, including a bias term, and passed through an activation function is associated with an error that represents the distance from the output to the expected prediction values. Neural nets adjust weights to reduce the error through algorithms like back-propagation (1, 42), but discussion of such concepts is beyond the scope of this article. If we imagine a neural net with four features as inputs, then going through a single hidden layer with three nodes (Figure 3C), the equation to calculate the output would be the following.

**Equation 5**. Deep neural network equation using tanh non-linearity.


$$\begin{array}{l} ŷ=v{\left[0\right]}^{*}h\left[0\right]+v{\left[1\right]}^{*}h\left[1\right]+v{\left[2\right]}^{*}h\left[2\right]+b \end{array}$$

v: weights between the hidden layer and the output.

h: intermediate values stored in neurons of the hidden layer.

h is calculated as:


$$h\left[i\right]=\;\text{tanh}\left(\left(\overset{\left(H-1\right)}{\underset{\left(i=0\right)}{\sum }}\overset{\left(N-1\right)}{\underset{\left(j=0\right)}{\sum }}w\left[i,j\right]\right)+b\left[i\right]\right)$$

H: number of nodes in the hidden layers.

N: number of features

w: weight between the input and the nodes in the hidden layer.

When we applied a multilayer perceptron to the classification problem on the hepatitis dataset, we achieved an accuracy of 84.6%. A disadvantage associated with neural networks is that interpretation of the model is quite troublesome. In our example, we applied a neural network and calculated and deployed the weights associated with such a neural network in a heat map (Figure 3D); results are far from clear. A statement that can be made is that features with smaller weights are less important for the model (their influence on the target variable is less significant).

Figure 2F presents the classification results achieved by several algorithms. Logistic regression achieved an *R*^2^ of 90%, accuracy of other algorithms (k-NN, support vector classifier, SVC; stochastic gradient descent classifier, SDGC; random forest classifier, RFC and multi-layer perceptron classifier, MLPC) ranged between 82 and 85%.

**Figure 4:**
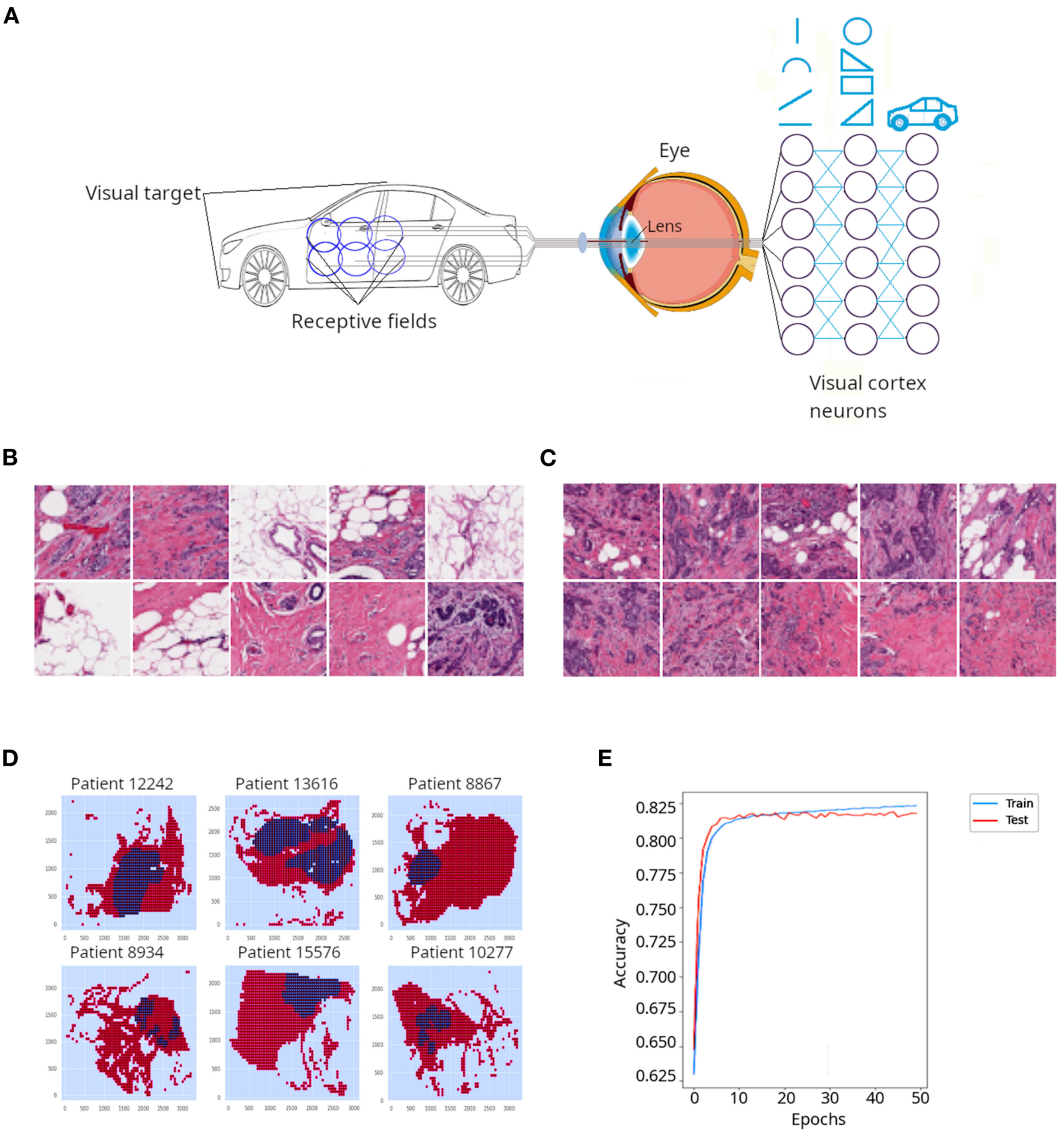
Application of a convolutional neural network (CNN) to classify cancer tissue. **(A)** CNNs are emulations of sets of neurons that detect individual overlapping visual sections called receptive fields. Such neurons detect simple features of the object, such as lines and arcs. Deeper neuronal layers detect more complex shapes derived from the initial elements and progressively the whole object is resolved. **(B)** Representative patches from the invasive ductal carcinoma (IDC) tissue sections described in Cruz-Roa et al. (46) and classified as non-cancerous tissue by a pathologist. **(C)** Representative patches from the invasive ductal carcinoma (IDC) tissue sections classified as cancerous tissue by a pathologist. In **(B,C)**, darker regions correspond to nuclei stained with hematoxylin, which appear denser in **(C)** probably due to increased cell proliferation in cancerous tissues. **(D)** Digital reconstruction of tissue sections from individual patches in six different patients. Red regions represent non-cancerous tissue, while blue regions represent cancerous tissue. **(E)** Accuracy for training and test data sets obtained when a CNN was applied to the IDC dataset.

Another type of neural nets are convolutional neural networks (CNNs or ConvNets), which are often applied to the field of computer vision to conduct image classification. CNNs were inspired by the organization of the visual cortex of the human brain (43). In their seminal work on cats and monkeys, Hubel and Wiesel (44, 45) determined that the individual neurons in the visual cortex were responsible for perceiving only a small portion of the visual field and the tiling of many overlapping visual subfields acquired by many neurons creates complex images. As illustrated in Figure 4A, when the brain attempts to perceive the image of a car, a whole image will be the composite of many subfields that observe individual overlapping sections of the car. The authors also discovered a high level of diversity and specialization among neurons of the visual cortex: some of them were dedicated to the perception of simple geometric patterns like lines and arcs, while other higher-level neurons were able to perceive more complex patterns, derived from the combination of lower level patterns (Figure 4A). For such breakthrough discoveries, Hubel and Wiesel won the Nobel prize for Physiology and Medicine in 1981.

Although CNNs were conceived in the 1980's (47, 48), they remained in the shadows because of their initial inability to scale up: they needed a lot of images and hence computer resources to perform considerably well. However, it should not be surprising that emulating the primary visual pathway of the human brain has been troublesome, after all it took nature 500 million years to evolve such a system (49). Recent advances in computer power and the exponential accumulation of images in diverse realms of research and technology revived interest in CNNs (50, 51).

As said above, CNNs function in a highly hierarchical manner. Initially, a CNN layer starts detecting lines and arcs. This information is passed to the next convolutional layer, which detects combinations of edges and corners. Eventually, the deeper layers of the convolutional network are able to detect complex patterns, like faces, cars, cancerous tissues, etc. They are called convolutional because to transfer information across layers of the network, mathematical convolution is used. Convolution refers to the combination of two functions to produce a third function; or more simply put, two sources of information are merged into a single function (52).

In a conventional neural net, the neurons of a hidden layer are fully connected to all neurons in the contiguous layer, and finally a fully-connected output layer provides the predictions of the network (53). In a CNN, layers are three dimensional (width, height, and depth), and more importantly, the neurons in a layer are not connected to all neurons in the next layer, but instead to only a small number of them. The output could be a class or a vector of probability of classes. CNNs processing starts with feature extraction; for that, a filter or kernel (of size equal to the receptive field) is slid along the full image to create a feature map, which is the sum of convolutions. Finally, a classification procedure is applied (43).

In order to illustrate CNNs, we used breast cancer (invasive ductal carcinoma, IDC) sections that had been classified by a pathologist (46). The authors divided the slides' pictures into 100 × 100 pixels non-overlapping patches, which resulted in more than 270,000 patches. A patch was considered positive if at least 80% of the patch was contained inside the cancerous annotated region, or negative otherwise. Examples of negative and positive patches are presented in Figures 4B,C, respectively. Digital reconstructions of sections from its constituent patches are depicted in Figure 4D. Because the IDC dataset is largely unbalanced, i.e., many negative and few positive patches, we extracted a subsample with equal number of positive and negative patches (~13,000 patches in each class), in order to be able to evaluate the performance of our CNN using accuracy as a metric (accuracy is not a reliable metric for unbalanced datasets). Although we run the model for 50 epochs (iterations) it quickly (around epoch 20) plateaued between 81 and 82% (Figure 4E). Thus, a relatively simple implementation of a CNN yielded satisfactory classification of cancerous and non-cancerous breast tissues.

In addition to conventional (feed forward) neural nets and CNNs, there exist other types of more complex neural nets not discussed here, including long short-term memory (LSTM) (50, 54) and Kohonen's self-organizing maps (SOM) (55), which find applications in, for example, protein folding (54) and hematopoietic differentiation (56), respectively.

In summary, supervised ML approaches have great potential in biomedical research because supervision of the model performance with real clinical data provides confidence for making decisions about treatment of patients. The emphasis of this report is just to provide a general overview of how ML approaches work, providing enough details for the reader to gain a good grasp about the underlying methods but without entering into particular details of algorithms or unnecessary mathematical explanations.

### Unsupervised Learning

#### Clustering and Ordination

There are two families of techniques in unsupervised learning: clustering and dimensionality reduction (ordination). Clustering aims at partitioning data into constituents, usually based on distance among samples. The basic idea is that data points in the same cluster have similar properties, and are more different from data points in other clusters. There exist a variety of clustering algorithms—including agglomerative clustering, DBSCAN, KMeans, Birch clustering, Gaussian mixture model, spectral clustering, etc. (1, 57). Their efficiency and reliability depends on the distribution of the dataset under analysis; this means no single algorithm performs best on all tasks This is why deciding on the best model needs to be determined empirically. Also, some algorithms scale better than others; for example, algorithms that compute pairwise similarities among all samples do not work well for large datasets.

Instead of defining each clustering algorithm, we will illustrate some of them with a simulated example. We generated 10,000 random instances (each a pair of values) grouped into three classes, each of them with a normal distribution (Supplementary Figure 3A). To evaluate the performance of clustering methods, the Silhouette score may be used. The Silhouette score is a metric to estimate the robustness of clustering, whose value ranges from−1 to 1, with higher values associated with clusters integrated by similar samples, while low values indicate that clusters contain heterogenous samples (58, 59). We applied a series of clustering algorithms on our simulated 3-clusters dataset (Supplementary Figures 3B–F), and then colored each according to the cluster it was assigned to by the clustering algorithm. As seen, all clustering algorithms tested achieved very similar results, faithfully reflecting clustering in the original dataset. KMeans clustering produced a Silhouette score (0.506) that was slightly higher than the other methods: Spectral clustering (0.492), Gaussian mixture clustering (0.484), Birch clustering (0.479) and Agglomerative clustering (0.471). As said above, no clustering method is the best, but KMeans is often a good starting point.

The number of input variables or features describing an instance is called its dimensionality. Techniques for dimensionality reduction are often used for visualization. Dimensionality reduction techniques use linear algebra, projection methods and autoencoders (see below for a brief discussion on autoencoders). Many ordination techniques were developed in the context of population ecology, where the interest was to know the relationship among groups (e.g., species) in a community (60, 61). Popular ordination approaches include distance-based techniques like Principal coordinates analysis (PCoA) and Non-metric multidimensional scaling (MDS), Eigen vector gradient analysis like Principal component analysis (PCA) and Correspondence analysis (CA), and manifold learning like autoencoders, isomaps and t-distributed stochastic neighbor embedding (t-SNE) (1, 57, 62).

Because those techniques have been widely used in biomedical research for quite some time (60, 61, 63, 64), they will not be discussed in detail here. However, we will illustrate t-SNE, PCA and MDS with real data. In a nutshell, t-SNE derives a probability distribution in the high-dimensional space using Euclidean distances between objects and a similar distribution in the low-dimensional space, trying to minimize the Kullback-Leibler divergence between the two probability distributions (65–67). The most important parameter of a t-SNE algorithm is the perplexity, which controls the width of the Gaussian kernel used to compute similarities between samples, and hence to control the number of nearest neighbors associated with a specific data point (68). Nevertheless, t-SNE is criticized for not preserving global structure of data, which may be critical for some practical applications (69). PCA is a multivariate statistical technique developed at the very beginning of the twentieth century by none other than Pearson (70). The central tenet in PCA is to reduce dimensionality of multidimensional datasets with interrelated features, to be able to visualize data in a low-dimensional space that contain most of the variance of such a dataset. This is achieved by transforming the original feature into uncorrelated, orthogonal, principal components (63, 71). MDS is a representation and dimensionality reduction technique that maps instances into a low-dimensional space in a way that attempts to preserve the relative distances between instances. Samples that are more similar will be represented near to each other, while different samples will be represented apart (72, 73). Mathematically, it transforms, using Eigenvalues decomposition, a dissimilarity matrix (distance between samples) into a coordinate matrix while minimizing a loss function; in other words, trying to preserve the original distances between samples (64).

**Figure 5:**
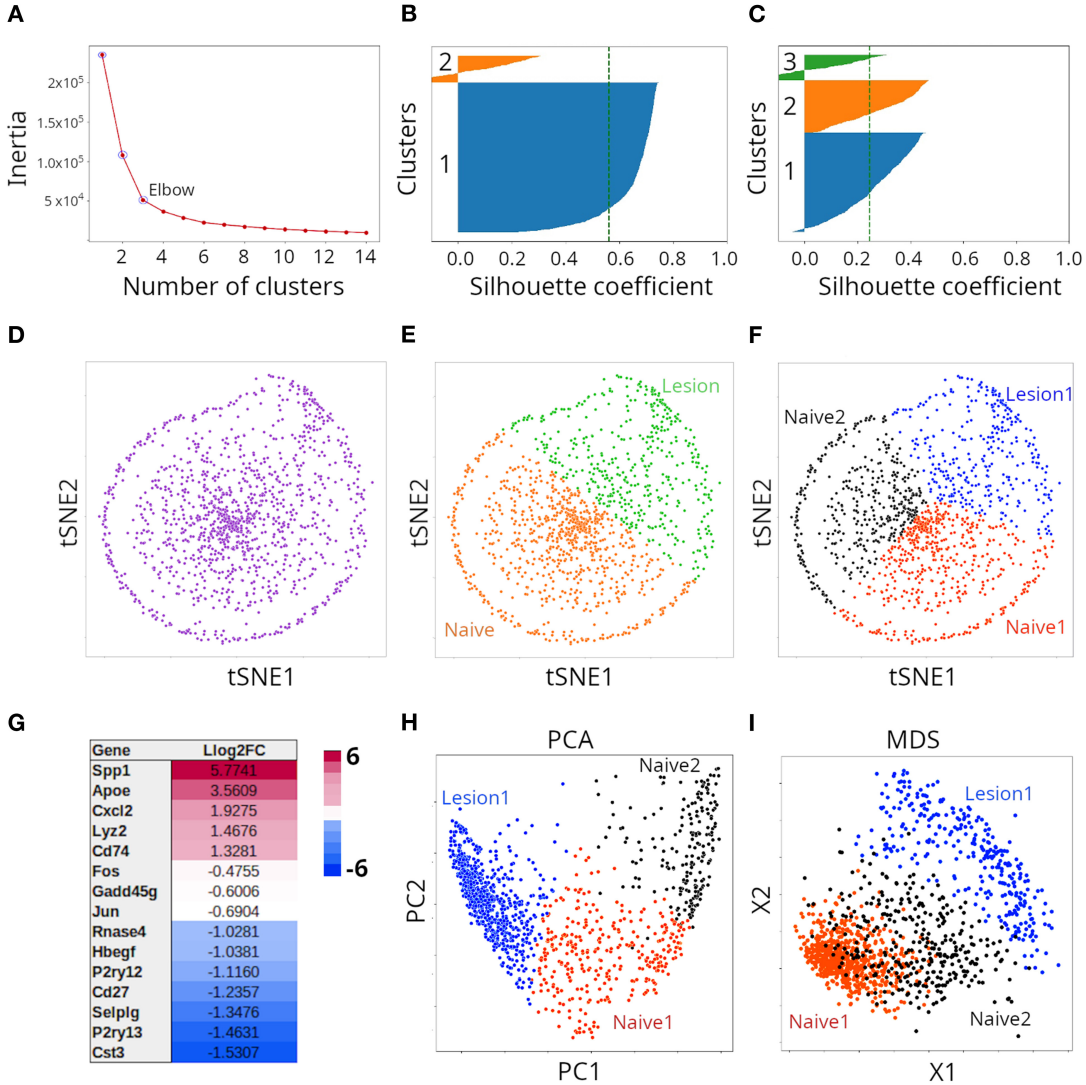
Clustering of microglia single cell transcriptomes using tSNE, PCA or MDS. **(A)** Determination of optimal number of clusters through K-means clustering. Validation of cluster number (n) with the Silhouette method when two **(B)** or three **(C)** clusters were considered. **(D)** Transcriptome samples clustered with tSNE (*n* = 2) and colored with a single color, or with two colors **(E)**. Such clusters correspond to naive and lesion cells. **(F)** Transcriptome samples clustered with tSNE (*n* = 3) and colored with three colors. Such clusters correspond to two naive and one lesion groups of cells. **(G)** Representative differentially expressed genes between naive and lesion cells (see **E**), were in agreement with (8). **(H)** Transcriptome samples clustered by PCA or with MDS **(I)**.

To provide a more practical illustration of both clustering and ordination techniques, we analyze here, in an agnostic way, transcriptomics single-cell data from (8). The dataset contains gene abundance derived by 10X Chromium technology from 1,291 individual microglia from mice with injury in their spinal cord, as well as naive animals. In order to select the optimal number of clusters defined by the dataset, we used the elbow method of the KMeans clustering algorithm. In this method, the algorithm is fit to a range of cluster numbers (k). For each k, we compute the inertia, which is the sum of squared distances of instances to the closest cluster center; in other words, how far away points within a cluster are located. When plotting the inertia as a function of the number of classes, we typically see an arm-like shape; we can then use the point of inflection of the curve (elbow) as an indicator of best fit of the model. In our case, the elbow was located at three clusters (Figure 5A). However, because we had the knowledge presented by Plemmel et al. in their paper, we knew that two (naive vs. injury), three (two naive vs. one major injury groups) or five (two naive vs. three injury groups) clusters were correct forms to partition the dataset. Silhouette coefficients of KMeans clusters suggested partition of data points into two (Figure 5B) or three (Figure 5C) clusters. For graphical representation, we initially chose the t-SNE method. As seen in Figure 5D, t-SNE deployed instances into a somewhat circular distribution; however, the specific shape of the t-SNE plot is highly dependent on the data transformation method used prior to t-SNE and the value of perplexity chosens. We applied the StandardScaler method of Scikit-learn and a perplexity of 30, which is the default value in most t-SNE implementations. Because we knew that the cells represented subpopulations from mice with or without injury in their spinal cord, we initially clustered the data points population into two clusters (Figure 5E; orange and green clusters) and subsequently into three clusters (Figure 5F; red, black and blue clusters). Plemmel and collaborators reported that cells in lesion 1 exhibited higher expression of the genes *Apoe, Spp1, Cxcl2, Lyz2*, and *Cd74*. Accordingly, we conducted differential expression analysis, and found that indeed all those five genes were differentially expressed when the two naive clusters (together) were compared against lesion 1 samples (Figure 5G). Thus, combining KMeans clustering and t-SNE, we were able to recapitulate the results reported by Plemmel and collaborators for the major lesion samples vs. the naive ones. To further test the reliability of our clustering, we subjected such classification to supervised ML. We applied gradient boosting classification and could indeed predict labels of the two clusters (Figure 5E) with an accuracy of 98% and labels of the three clusters (Figure 5F) with an accuracy of 94%. For comparison, we also clustered the same data using PCA (Figure 5H) and MDS (Figure 5I) and found that both methods effectively separated the same three clusters but separation of clusters was less clear than in the case of t-SNE. When we applied a regularized logarithmic transformation to the data, prior to ordination, it substantially improved the resolution of clusters for t-SNE, but had the opposite effect for PCA and MDS (Supplementary Figure 4). We did not explore this in more detail.

#### Autoencoders

Autoeconders are typically artificial neural networks (ANNs) used for representation learning. Representation learning is a technique that allows a model to learn features essential for accomplishing a specific analytical task. An autoencoder learns to compress data and also has the capability to reconstruct such data to generate a representation that attempts to be as similar as possible to the original data. These two tasks are accomplished by two submodels: the encoder (recognition network) and the decoder (generative network), respectively (74–76). The encoder can be viewed as a filter that selects some of the most relevant features that are sufficient to represent the data in a compressed format (fewer dimensions). Between them is the compressed code that is able to regenerate the data and is also known as latent-space representation (74). However, although the decoder aims at reconstructing the original data, it only uses the information contained in the code. The difference between the original and the regenerated data is called the error, which is estimated by a loss function. Because autoencoders remove noise to generate the compressed representation of the data, they naturally reduce dimensions. There are several types of autoencoders, including undercomplete, stacked, sparse, convolutional, contractive and variational ones, among others. For a detailed description of such approaches, see (19, 75).

For the sake of clarity, we reproduce here an example presented by Géron (19) that helps to understand the encoding process. Consider two vectors of numbers:

[40, 27, 25, 36, 81, 57, 10, 73, 19, 68]

[50, 25, 76, 38, 19, 58, 29, 88, 44, 22, 11, 34, 17, 52, 26, 13, 40, 20]

The second vector, despite being longer, is easier to encode than the first one, because it contains a pattern. Every even number is followed by its half (50 by 50/2 = 25), while odd numbers are followed by its triple plus one (25 by 25 × 3+1 = 76). Thus, to learn the second sequence of numbers, the encoder only has to deduce these two rules, the first number in the series, and the length of the series of numbers. The first vector would be difficult to compress. Thus, autoencoders work better when elements in a data set contain patterns and poorly when they are independent from each other. Thus, the task of the autoencoder is to detect correlations between input features (19).

**Figure 6:**
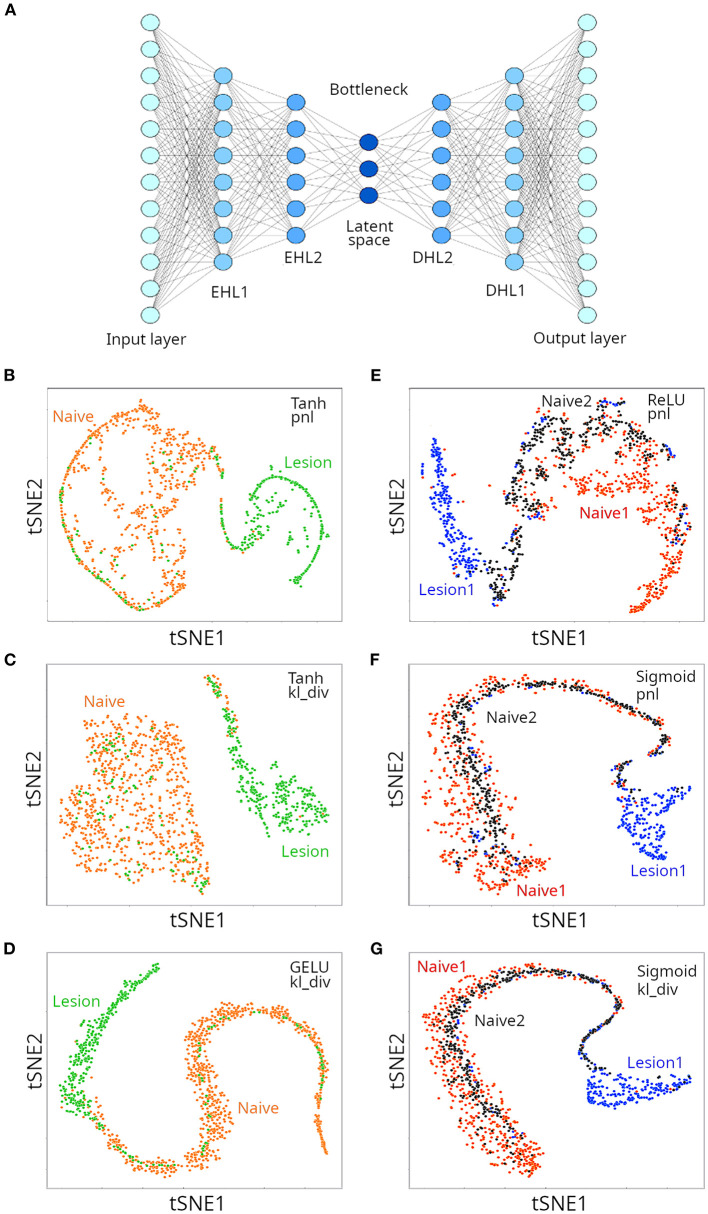
Clustering of microglia single cell transcriptomes using autoencoders. **(A)** Cartoon depicting an autoencoder neural network. When aiming at discriminating between two clusters, we used Tanh **(B,C)** and GELU **(D)** as activation functions for hidden layers, and either Poisson NLL (pnl) **(B)** or Kullback Liebler divergence (kv_div) **(C,D)** loss functions. When aiming at discriminating between three clusters, either ReLU **(E)**, or sigmoid **(F,G)** were used as activation functions. Loss functions are also indicated **(E–G)**.

The architecture of an autoencoder is similar to the ANNs (multi-layer perceptrons) presented in Figure 3, with two caveats: (i) the number of neurons in the output layers is the same as the number of inputs (features), because the autoencoder tries to regenerate the original data; and (ii) for undercomplete autoencoders, the hidden layers have fewer neurons than inputs, that force the autoencoder to select only the most relevant features in a compressed representation (Figure 6A). We applied undercomplete autoencoders to assess whether a better visual representation of the suspected two or three clusters could be obtained for the microglia single cell transcriptomics data. As mentioned above, among critical parameters of an ANN are the activation and loss function used, which affect performance of the model in a data-type dependent manner. When trying to separate two clusters, we used the Tanh and GELU activation functions (for hidden layers) and the Poisson NLL loss (pnl) and the Kullback Liebler divergence (kl_div) loss functions. We tested three configurations, and found each was successful in separating naive from lesion cells (Figures 6B–D). In all cases, the decoder loss was smaller than 10% (loss < 0.1), and the lowest error (loss) was reported for the configuration GELU-kl_div. We then run a gradient boosting classifier (GBC) to train a model that could classify instances into the appropriate original labels from the compressed representation of data in the latent space of the autoencoder. The resulting GBC model was able to classify the compressed representation of two clusters with an accuracy of 94%. We then tried to separate three clusters in the single cell population, using the same loss functions, combined with the ReLU or GELU activation functions. Three configurations tested provided satisfactory separation of the three expected clusters (Figures 6E–G), but the lowest loss was obtained for the configuration ReLU-pnl. A GBC was able to classify data points in the latent space of the autoencoder with an accuracy of 88%. The autoencoder compressed the 12,138 features (transcripts) into 40 compressed features. Thus, although using an autoencoder to generate low-dimensional representation of the single-cell RNAseq data slightly reduced the classification accuracy with a GBC model, the visual representation had higher resolution and allowed better discrimination of the clusters. A few instances corresponding to the lesion cluster were spread out away from the corresponding cluster. It is possible that those data points correspond to the small lesion clusters reported by Plemel et al. (8).

#### Anomaly Detection

In classical statistics, an outlier can be defined as an observation that lies at an unexpected distance from the rest of observations in a random sample from the same population. Assessment of such observations usually involves graphical methods like scatter or box plots, and were historically identified and removed from datasets. ML also offers methods to detect outliers and refers to them as anomalies (77–79). Essentially, a model is trained with “normal” instances, and learns to identify instances that deviate from such a subset (19, 80). Initially, those samples were removed for subsequent analysis, but more recently they are studied as cases that could represent critical stages of a phenomenon under study. Such methods have seen applications in many fields, including biomedical research. Examples are detection of anomalous signals from body sensors, or detection of cancer cells in micrographs of tissue from cancer patients in early stages, and many more (66, 81–83).

Finally, we would like to mention a very exciting class of ML frameworks dubbed generative adversarial networks (GANs) where supervised and unsupervised deep learning notions converge. Here, an unsupervised generative model is trained using two neural networks that compete in a zero-sum game (84). Informed by statistics from the training dataset, a generative model learns to create new instances, while a discriminator model attempts to differentiate between instances generated by the contesting model and the real instances from the actual training dataset (50, 75, 84). The two models update each other until the generator model fools the discriminator model half of the time (85). GANs can be applied to different fields in biomedical research, including clinical image processing (through CNNs), prediction of disease outcome, and modeling of cell differentiation from single cell RNAseq data (86–88).

## Concluding Remarks

Machine learning (ML) comprises a vast collection of computational methods that attempt to extract patterns from data, then use those patterns to derive mathematical models that are able to generalize the learned rules on unseen data. In other words, it generates artificial intelligence. In biomedical research, supervised and unsupervised ML techniques have been applied for decades, including regression analyses and clustering (1, 32, 36, 57, 64). However, with the exponential increase in computer power and data availability, ML has gained renewed impetus, especially in the area of deep learning. Application of modern ML approaches extends across many areas in biomedical research (14) ranging from analysis of clinical images, to stratification of patients into most promising therapeutic interventions, to drug discovery, and robotics, just to mention a few. Our goal was to bring an updated and simplified perspective of ML for non-experts in hope that members of the biomedical research community would realize the opportunities that ML offers for basic and applied researchers.

## Data Availability Statement

Publicly available datasets were analyzed in this study. This data can be found at: https://www.kaggle.com

## Author Contributions

JJ and RG: design of study, analysis of data, and preparation of manuscript. Both authors contributed to the article and approved the submitted version.

## Conflict of Interest

The authors declare that the research was conducted in the absence of any commercial or financial relationships that could be construed as a potential conflict of interest.

## Publisher's Note

All claims expressed in this article are solely those of the authors and do not necessarily represent those of their affiliated organizations, or those of the publisher, the editors and the reviewers. Any product that may be evaluated in this article, or claim that may be made by its manufacturer, is not guaranteed or endorsed by the publisher.

## Supplementary Material

Supplementary Figure 1 Graphical description of the hepatitis dataset used to illustrate supervised machine learning algorithms.

Supplementary Figure 2 **(A)** Decision tree obtained when the DTC algorithm was applied to the hepatitis dataset. **(B)** Comparison of decision surfaces of a single decision tree and a random forest applied for classification of survival of the hepatitis dataset (only albumin and bilirubin were used for creation of such plots).

Supplementary Figure 3 Illustration of clustering methods on simulated data. **(A)** 10,000 random points clustered into three clusters were generated. Such random points were reorganized into three clusters using agglomerative clustering **(B)**, Birch clustering **(C)**, KMeans clustering **(D)**, spectral clustering **(E)** and Gaussian mixture clustering **(F)**. The Silhouette score is included in each case.

Supplementary Figure 4 tSNE **(A)**, PCA **(B)**, and MDS **(C)** when the microglia single cell transcriptome data was normalized using a regularized logarithmic transformation.

## References

[1] Hastie T Tibshirani R Friedman J. The Elements of Statistical Learning: Data Mining, Inference, and Prediction, Second Edition. New York, NY: Springer Science & Business Media (2009).

[2] Jordan MI Mitchell TM. Machine learning: Trends, perspectives, and prospects. Science. (2015) 349:255–60. 10.1126/science.aaa8415 26185243

[3] Müller AC Guido S. Introduction to Machine Learning with Python: A Guide for Data Scientists. Sebastopol, CA: O'Reilly Media, Inc. (2016).

[4] Ayodele TO. Types of machine learning algorithms. New Adv Mach Learn. (2010) 3:19–48. 10.5772/9385

[5] Berry MW Mohamed A Yap BW. Supervised and Unsupervised Learning for Data Science. Cham: Springer Nature (2019). 10.1007/978-3-030-22475-2

[6] Walker A Surda P. Unsupervised learning techniques for the investigation of chronic rhinosinusitis. Ann Otol Rhinol Laryngol. (2019) 128:1170–6. 10.1177/0003489419863822 31319675

[7] Sindhu Meena K Suriya S. A survey on supervised and unsupervised learning techniques. In: Proceedings of International Conference on Artificial Intelligence, Smart Grid and Smart City Applications (Springer International Publishing) (2020). 10.1007/978-3-030-24051-6_58

[8] Plemel JR Stratton JA Michaels NJ Rawji KS Zhang E Sinha S . Microglia response following acute demyelination is heterogeneous and limits infiltrating macrophage dispersion. Sci Adv. (2020) 6:eaay6324. 10.1126/sciadv.aay6324 31998844 PMC6962036

[9] Francois-Lavet V Henderson P Islam R Bellemare MG Pineau J. An Introduction to Deep Reinforcement Learning. arXiv [cs.LG]. (2018). Available online at: http://arxiv.org/abs/1811.12560

[10] Majumder A. Introduction to reinforcement learning. Deep Reinforce. Learn. Unity. (2021) 2021:1–71. 10.1007/978-1-4842-6503-1_1

[11] Sutton RS Barto AG. Reinforcement Learning, Second edition: An Introduction. Cambridge, MA: MIT Press (2018).

[12] Sutton RS McAllester DA Singh SP Mansour Y. Policy Gradient Methods for Reinforcement Learning With Function Approximation. Cambridge, Massachusetts (1999). Available online at: https://proceedings.neurips.cc/paper/1999/file/464d828b85b0bed98e80ade0a5c43b0f-Paper.pdf (accessed May 20, 2021).

[13] Chuang LY Tsai JH Yang CH. Operon prediction using particle swarm optimization and reinforcement learning. In: 2010 International Conference on Technologies and Applications of Artificial Intelligence (2010). 10.1109/TAAI.2010.65

[14] Mahmud M Kaiser MS Hussain A Vassanelli S. Applications of deep learning and reinforcement learning to biological data. IEEE Trans Neural Netw Learn Syst. (2018) 29:2063–79. 10.1109/TNNLS.2018.2790388 29771663

[15] Graesser L Keng WL. Foundations of Deep Reinforcement Learning: Theory and Practice in Python. Addison-Wesley Professional (2019).

[16] Petersen BK Yang J Grathwohl WS Cockrell C Santiago C An G . Deep reinforcement learning and simulation as a path toward precision medicine. J Comput Biol. (2019) 26:597–604. 10.1089/cmb.2018.0168 30681362 PMC6590719

[17] McCorduck P. Machines Who Think: A Personal Inquiry into the History and Prospects of Artificial Intelligence. Canada: CRC Press (2004). 10.1201/9780429258985

[18] Okfalisa O Gazalba I Mustakim Reza NGI. Comparative analysis of k-nearest neighbor and modified k-nearest neighbor algorithm for data classification. In: 2017 2nd International conferences on Information Technology, Information Systems and Electrical Engineering (ICITISEE). Yogyakarta (2017). 10.1109/ICITISEE.2017.8285514

[19] Géron A. Hands-On Machine Learning with Scikit-Learn, Keras, TensorFlow: Concepts. Tools, and Techniques to Build Intelligent Systems. Sebastopol, CA: O'Reilly Media, Inc. (2019).

[20] Diaconis P Efron B. Computer-intensive methods in statistics. Sci. Am. (1983) 248:116–31. 10.1038/scientificamerican0583-116

[21] Cestnik B Kononenko I Bratko I. A knowledge-elicitation tool for sophisticated users. In: Proceedings of the 2nd European Conference on European Working Session on Learning EWSL'87. Sigma Press (1987).

[22] Baskaya B. Statistical Analysis of Decision Trees. Long Beach, CA: California State University (2011).

[23] Kingsford C Salzberg SL. What are decision trees? Nat Biotechnol. (2008) 26:1011–3. 10.1038/nbt0908-1011 18779814 PMC2701298

[24] Kotsiantis SB. Decision trees: a recent overview. Artif Intell Rev. (2013) 39:261–83. 10.1007/s10462-011-9272-4

[25] Dahan H Cohen S Rokach L Maimon O. Proactive Data Mining with Decision Trees. New York, NY: Springer Science & Business Media. (2014). 10.1007/978-1-4939-0539-3

[26] Slocum M. Decision making using id3 algorithm. Insight: River Acad J. (2012) 2012:8. Available online at: https://www2.rivier.edu/journal/ROAJ-Fall-2012/J674-Slocum-ID3-Algorithm.pdf (accessed March 13, 2020).

[27] Yang S Guo JZ Jin JW. An improved Id3 algorithm for medical data classification. Comput Electr Eng. (2018) 65:474–87. 10.1016/j.compeleceng.2017.08.005

[28] Natekin A Knoll A. Gradient boosting machines, a tutorial. Front Neurorobot. (2013) 7:21. 10.3389/fnbot.2013.00021 24409142 PMC3885826

[29] Ke G Meng Q Finley T Wang T Chen W Ma W . Lightgbm: A highly efficient gradient boosting decision tree. Adv Neural Inf Process Syst. (2017) 30:3146–54. Available online at: https://proceedings.neurips.cc/paper/2017/file/6449f44a102fde848669bdd9eb6b76fa-Paper.pdf (accessed August 05, 2020).

[30] Zhang Z Zhao Y Canes A Steinberg D Lyashevska O. Predictive analytics with gradient boosting in clinical medicine. Ann Transl Med. (2019) 7:152. 10.21037/atm.2019.03.29 31157273 PMC6511546

[31] Matloff N. Statistical Regression and Classification: From Linear Models to Machine Learning. Boca Raton, Fl: CRC Press (2017). 10.1201/9781315119588

[32] Montgomery DC Peck EA Geoffrey Vining G. Introduction to Linear Regression Analysis. Hoboken, NJ: John Wiley & Sons (2012).

[33] Jacobson SH. Optimal mean squared error analysis of the harmonic gradient estimators. J Optimiz Theory App. (1994) 80:573–90. 10.1007/BF02207781

[34] Ruder S. An Overview of Gradient Descent Optimization Algorithms. arXiv [cs.LG]. (2016). Available online at: http://arxiv.org/abs/1609.04747

[35] Menard S. Logistic Regression: From Introductory to Advanced Concepts and Applications. Thousand Oaks, CA: SAGE (2010). 10.4135/9781483348964

[36] Hosmer DW Lemeshow S Sturdivant RX. Applied Logistic Regression. John Wiley & Sons. (2013). 10.1002/9781118548387

[37] LeCun BY Hinton G. Deep learning. Nature. (2015) 521:436–44. 10.1038/nature14539 26017442

[38] McCulloch WS Pitts W. A logical calculus of the ideas immanent in nervous activity. Bull Math Biophys. (1943) 5:115–33. 10.1007/BF02478259 2185863

[39] Rosenblatt F. The perceptron: a probabilistic model for information storage and organization in the brain. Psychol Rev. (1958) 65:386–408. 10.1037/h0042519 13602029

[40] Agostinelli F Hoffman M Sadowski P Baldi P. Learning Activation Functions to Improve Deep Neural Networks. arXiv [cs.NE]. (2014). Available online at: http://arxiv.org/abs/1412.6830

[41] >Nwankpa C Ijomah W Gachagan A Marshall S. Activation Functions: Comparison of trends in Practice Research for Deep Learning. arXiv [cs.LG]. (2018). Available online at: http://arxiv.org/abs/1811.03378

[42] Buturovic LJ Citkusev LT. Back propagation and forward propagation. In: [Proceedings 1992] IJCNN International Joint Conference on Neural Networks. Baltimore, MD (1992). 10.1109/IJCNN.1992.227297

[43] Albawi S Mohammed TA Al-Zawi S. Understanding of a convolutional neural network. In: 2017 International Conference on Engineering and Technology (ICET). (2017). 10.1109/ICEngTechnol.2017.8308186

[44] Hubel DH Wiesel TN. Receptive fields of cells in striate cortex of very young. Visually inexperienced kittens. J Neurophysiol. (1963) 26:994–1002. 10.1152/jn.1963.26.6.994 14084171

[45] Hubel DH Wiesel TN. Receptive fields and functional architecture of monkey striate cortex. J Physiol. (1968) 195:215–43. 10.1113/jphysiol.1968.sp008455 4966457 PMC1557912

[46] Cruz-Roa A Basavanhally A. Automatic detection of invasive ductal carcinoma in whole slide images with convolutional neural networks. Med Imaging. (2014) 9041:3872. 10.1117/12.204387217645476

[47] LeCun B Denker JS Henderson D Howard RE. Hubbard W . Backpropagation applied to handwritten zip code recognition. Neural Comput. (1989) 1:541–51. 10.1162/neco.1989.1.4.541

[48] LeCun B Bengio Y. Convolutional networks for images, speech, time series. Handbook Brain Theory Neural Netw. (1995) 3361:1995.

[49] Suryanarayana SM Pérez-Fernández J Robertson B Grillner S. The evolutionary origin of visual and somatosensory representation in the vertebrate pallium. Nat Ecol Evol. (2020) 4:639–51. 10.1038/s41559-020-1137-2 32203472

[50] Alom MZ Taha TM Yakopcic C Westberg S Sidike P Nasrin MS. The History Began from AlexNet: A Comprehensive Survey on Deep Learning Approaches. arXiv [cs.CV]. (2018). Available online at: http://arxiv.org/abs/1803.01164

[51] Ismail Fawaz H Lucas B Forestier G Pelletier C Schmidt DF Weber J . InceptionTime: Finding AlexNet for time series classification. Data Min Knowl Discov. (2020) 34:1936–62. 10.1007/s10618-020-00710-y

[52] Pang Y Sun M Jiang X Li X. Convolution in convolution for network in network. IEEE Trans Neural Netw Learn Syst. (2018) 29:1587–97. 10.1109/TNNLS.2017.2676130 28328517

[53] Abdi H. A neural network primer. J Biol Syst. (1994) 2:247–81. 10.1142/S0218339094000179

[54] Conover M Staples M Si D Sun M Cao R. AngularQA: protein model quality assessment with LSTM networks. Comput Mathemat Biophys. (2019) 7:1–9. 10.1515/cmb-2019-0001

[55] Miljković D. Brief review of self-organizing maps. In: 2017 40th International Convention on Information and Communication Technology, Electronics and Microelectronics (MIPRO). (2017). 10.23919/MIPRO.2017.7973581

[56] Tamayo P Slonim D Mesirov J Zhu Q Kitareewan S Dmitrovsky E . Interpreting patterns of gene expression with self-organizing maps: methods and application to hematopoietic differentiation. Proc Natl Acad Sci USA. (1999) 96:2907–12. 10.1073/pnas.96.6.2907 10077610 PMC15868

[57] Saxena A Prasad M Gupta A Bharill N Patel OP Tiwari A . A review of clustering techniques and developments. Neurocomputing. (2017) 267:664–81. 10.1016/j.neucom.2017.06.053

[58] Aranganayagi S Thangavel K. Clustering categorical data using silhouette coefficient as a relocating measure. In: International Conference on Computational Intelligence and Multimedia Applications (ICCIMA 2007) Tamil Nadu, India (2007). 10.1109/ICCIMA.2007.328

[59] Dinh DT Fujinami T Huynh VN. Estimating the optimal number of clusters in categorical data clustering by silhouette coefficient. Commun Comp Inform Sci. (2019) 2019:1–17. 10.1007/978-981-15-1209-4_1

[60] Pielou EC. The Interpretation of Ecological Data: A Primer on Classification and Ordination. New York, NY: John Wiley & Sons (1984).

[61] Rohlf FJ James Rohlf F. The interpretation of ecological data: a primer on classification and ordination. E. C. Pielou. Q Rev Biol. (1985) 60:531. 10.1086/414660

[62] Popat SK Emmanuel M. Review and comparative study of clustering techniques. Int J Comp Sci Inform Technol. (2014) 5:805–12. Available online at: https://citeseerx.ist.psu.edu/viewdoc/download?doi=10.1.1.433.3348&rep=rep1&type=pdf (accessed March 3, 2021).

[63] Grossman GD Nickerson DM Freeman MC. Principal component analyses of assemblage structure data: Utility of tests based on eigenvalues. Ecology. (1991) 72:341–7. 10.2307/19389271938927

[64] Borg I Groenen PJF. Modern Multidimensional Scaling: Theory and Applications. New York, NY: Springer Science & Business Media (2005).

[65] Peluffo-Ordóñez DH Lee JA Verleysen M. Short review of dimensionality reduction methods based on stochastic neighbour embedding. In: Advances in Self-Organizing Maps and Learning Vector Quantization (Springer International Publishing) (2014). 10.1007/978-3-319-07695-9_6

[66] Linderman GC Rachh M Hoskins JG Steinerberger S Kluger Y. Efficient Algorithms for t-distributed Stochastic Neighborhood Embedding. arXiv [cs.LG]. (2017). Available online at: http://arxiv.org/abs/1712.09005

[67] Rogovschi N Kitazono J Grozavu N Omori T Ozawa S. t-Distributed stochastic neighbor embedding spectral clustering. In: 2017 International Joint Conference on Neural Networks (IJCNN). Anchorage, AK (2017). 10.1109/IJCNN.2017.7966046

[68] van der Maaten L. Visualizing Data using t-SNE. (2008). Available online at: https://www.jmlr.org/papers/volume9/vandermaaten08a/vandermaaten08a.pdf?fbclid=IwAR0Bgg1eA5TFmqOZeCQXsIoL6PKrVXUFaskUKtg6yBhVXAFFvZA6yQiYx-M (accessed May 17, 2021).

[69] Kobak D Berens P. The art of using t-SNE for single-cell transcriptomics. Nat Commun. (2019) 10:5416. 10.1038/s41467-019-13056-x 31780648 PMC6882829

[70] Pearson K. L III. On lines and planes of closest fit to systems of points in space. London, Edinburgh, and Dublin Philosophical Magazine and J Sci. (1901) 2:559–72. 10.1080/14786440109462720

[71] Jolliffe IT. Principal Component Analysis. Springer Science & Business Media (2013). 10.1002/9781118445112.stat06472

[72] Kruskal JB. Multidimensional Scaling. SAGE (1978). 10.4135/9781412985130

[73] Cox MAA Cox TF. Multidimensional scaling. In: Chen CH Härdle W Unwin A editors. Handbook of Data Visualization. Berlin: Springer Berlin Heidelberg (2008). 10.1007/978-3-540-33037-0_14

[74] Baldi P. Autoencoders, unsupervised learning, deep architectures. In: Proceedings of ICML Workshop on Unsupervised and Transfer Learning Proceedings of Machine Learning Research. (Bellevue, WA: PMLR) (2012) 37–49.

[75] Alpaydin E. Introduction to Machine Learning. Cambridge, MA: MIT Press (2020). 10.7551/mitpress/13811.001.0001

[76] Bank D Koenigstein N Giryes R. Autoencoders. arXiv [cs.LG] (2020). Available online at: http://arxiv.org/abs/2003.05991

[77] Noble CC Cook DJ. Graph-based anomaly detection. in Proceedings of the ninth ACM SIGKDD international conference on Knowledge discovery and data mining KDD '03. (New York, NY: Association for Computing Machinery) (2003) 631–36. 10.1145/956750.956831

[78] Song X Wu M Jermaine C Ranka S. Conditional Anomaly Detection. IEEE Trans Knowl Data Eng. (2007) 19:631–45. 10.1109/TKDE.2007.100927295638

[79] Chandola V Banerjee A Kumar V. Anomaly detection: A survey. ACM Comput Surv. (2009) 41:1–58.

[80] Mehrotra KG Mohan CK Huang H. Anomaly Detection Principles and Algorithms. Cham, Switzerland: Springer (2017).

[81] Hauskrecht M Valko M Kveton B Visweswaran S Cooper GF. Evidence-based anomaly detection in clinical domains. AMIA Annu Symp Proc. (2007) 319–23. PMC2655918 18693850

[82] Antonelli D Bruno G Chiusano S. Anomaly detection in medical treatment to discover unusual patient management. IIE Trans Healthc Syst Eng. (2013) 3:69–77. 10.1080/19488300.2013.787564

[83] Churová V Vyškovský R Maršálová K Kudláček D Schwarz D. Anomaly Detection Algorithm for Real-World Data and Evidence in Clinical Research: Implementation, Evaluation, and Validation Study. JMIR Med Inform. (2021) 9:e27172. 10.2196/27172 33851576 PMC8140384

[84] Goodfellow I Pouget-Abadie J Mirza M Xu B Warde-Farley D Ozair S . Generative adversarial networks. Commun ACM. (2020) 63:139–44. 10.1145/3422622

[85] Goodfellow I Pouget-Abadie J Mirza M Xu B Warde-Farley D Ozair S . Generative Adversarial Nets. In Ghahramani Z Welling M Cortes C Lawrence N Weinberger KQ editors, Advances in Neural Information Processing Systems, (Curran Associates, Inc.) (2014). Available online at: https://proceedings.neurips.cc/paper/2014/file/5ca3e9b122f61f8f06494c97b1afccf3-Paper.pdf

[86] Bing X Zhang W Zheng L Zhang Y. Medical Image Super Resolution Using Improved Generative Adversarial Networks. IEEE Access. (2019) 7:145030–8. 10.1109/access.2019.294486230472408

[87] Guan S Loew M. Using generative adversarial networks and transfer learning for breast cancer detection by convolutional neural networks. Medical Imaging 2019: Imaging Informatics for Healthcare, Research, and Applications. San Diego (2019). 10.1117/12.2512671

[88] Lan L You L Zhang Z Fan Z Zhao W Zeng N . Generative Adversarial Networks and Its Applications in Biomedical Informatics. Front Public Health. (2020) 8:164. 10.3389/fpubh.2020.00164 32478029 PMC7235323

