# Supplementary figures and images for: An Introduction to Machine Learning Approaches for Biomedical Research
Source: Front Med (Lausanne). 2021 Dec 16;8:771607. doi: 10.3389/fmed.2021.771607 (PMC8716730; doi:10.3389/fmed.2021.771607)

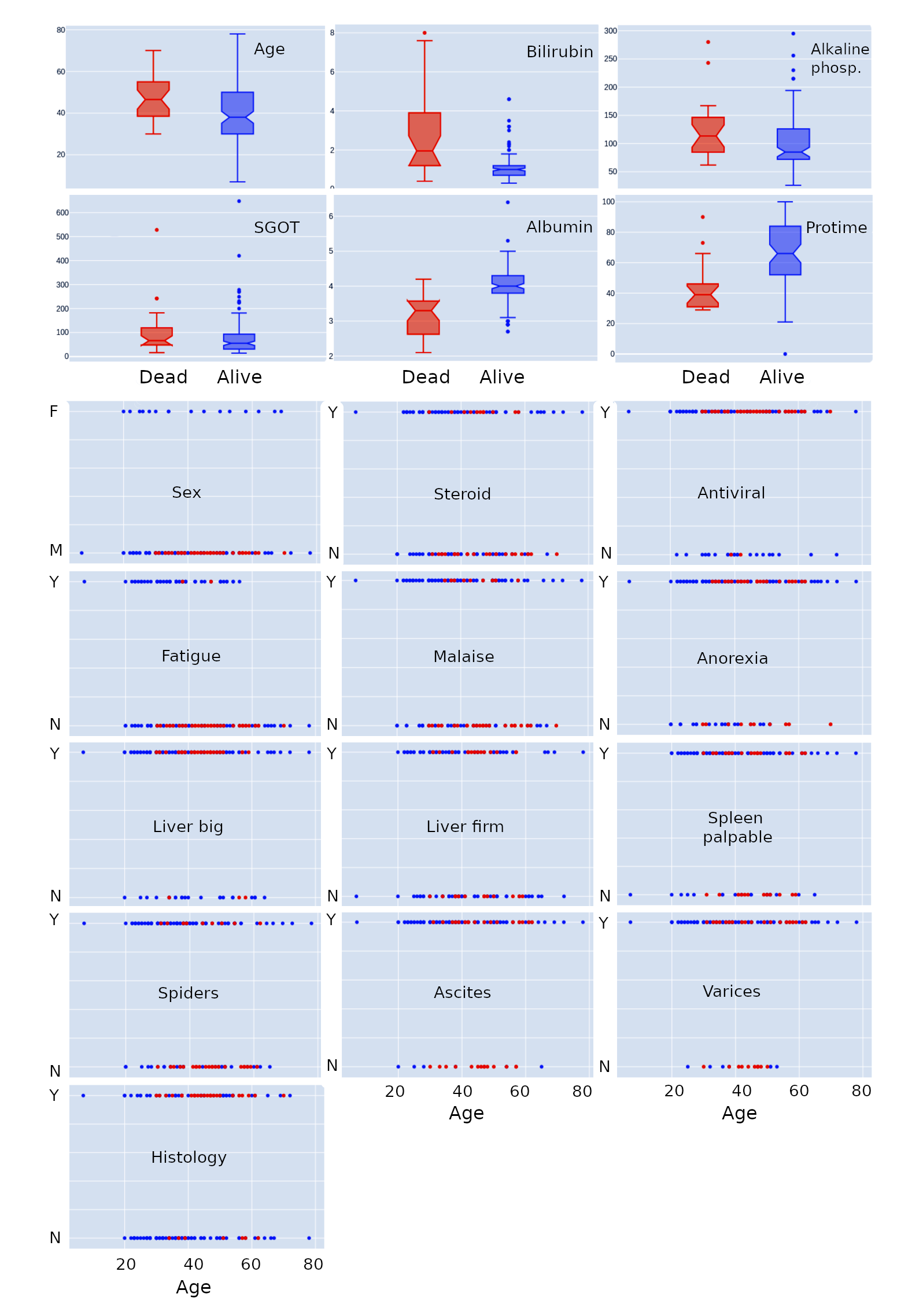

Supplement: Supplementary Figure 1 — Graphical description of the hepatitis dataset used to illustrate supervised machine learning algorithms. [file Image_1.TIFF]

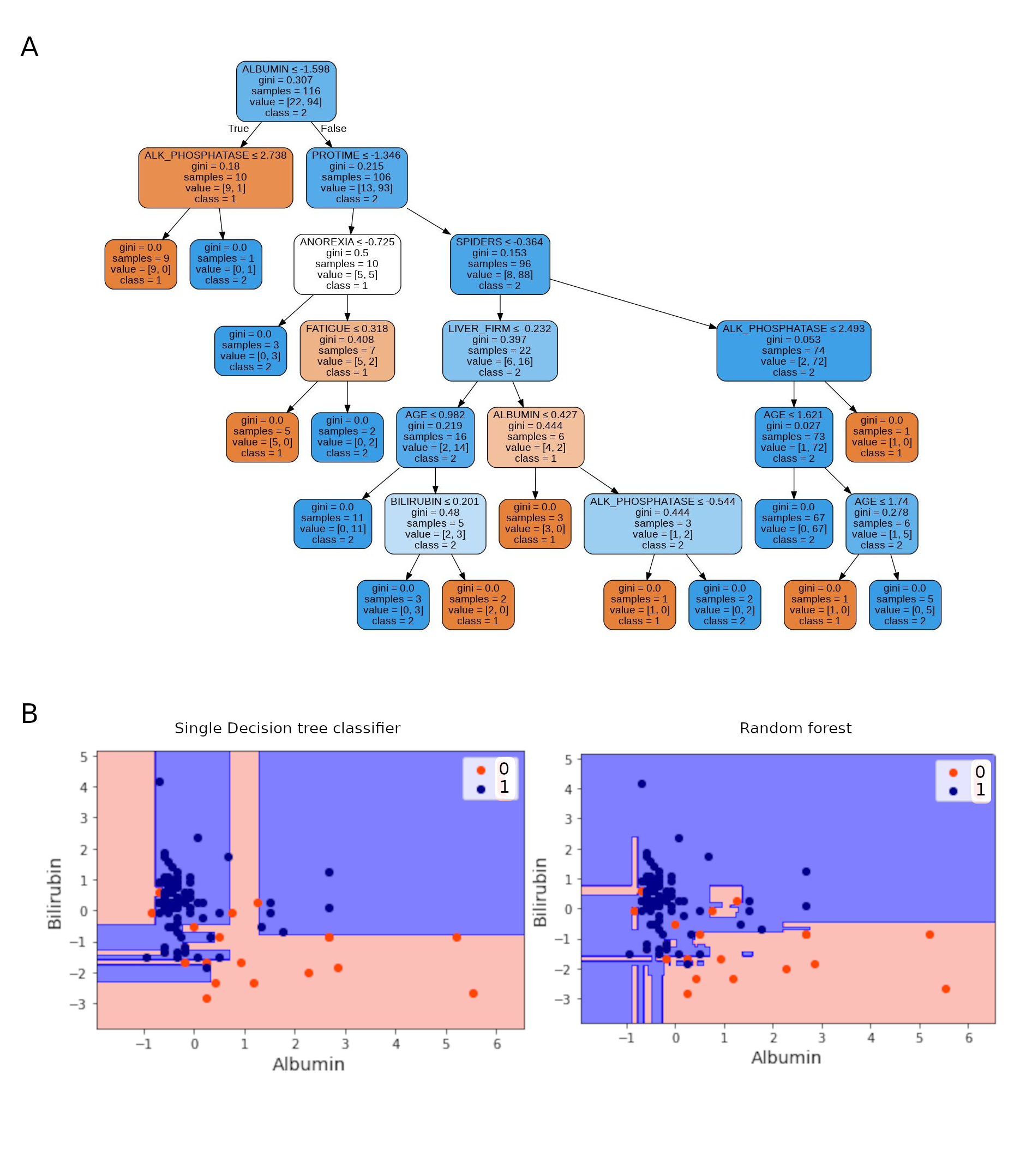

Supplement: Supplementary Figure 2 — (A) Decision tree obtained when the DTC algorithm was applied to the hepatitis dataset. (B) Comparison of decision surfaces of a single decision tree and a random forest applied for classification of survival of the hepatitis dataset (only albumin and bilirubin were used for creation of such plots). [file Image_2.TIFF]

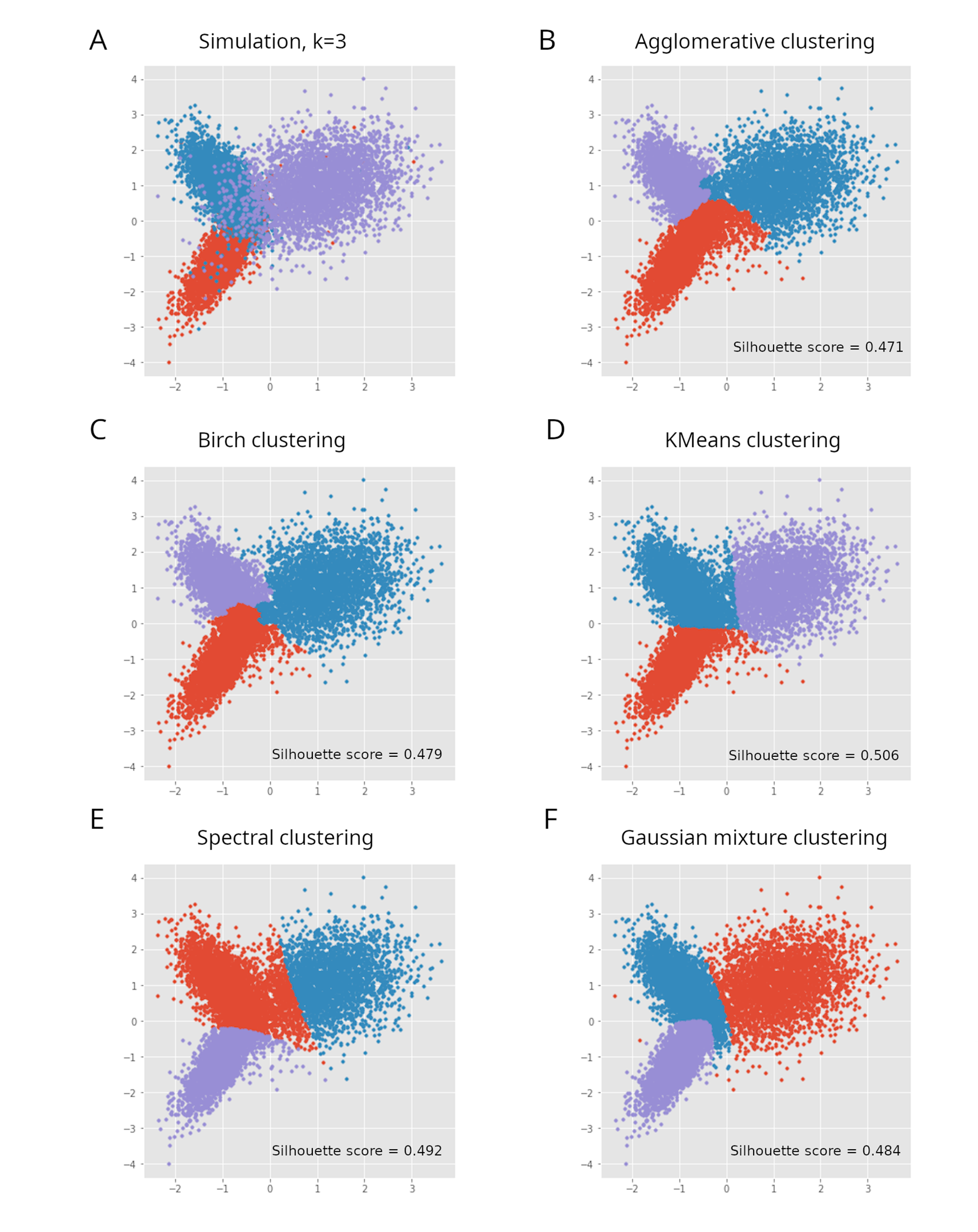

Supplement: Supplementary Figure 3 — Illustration of clustering methods on simulated data. (A) 10,000 random points clustered into three clusters were generated. Such random points were reorganized into three clusters using agglomerative clustering (B), Birch clustering (C), KMeans clustering (D), spectral clustering (E) and Gaussian mixture clustering (F). The Silhouette score is included in each case. [file Image_3.TIFF]

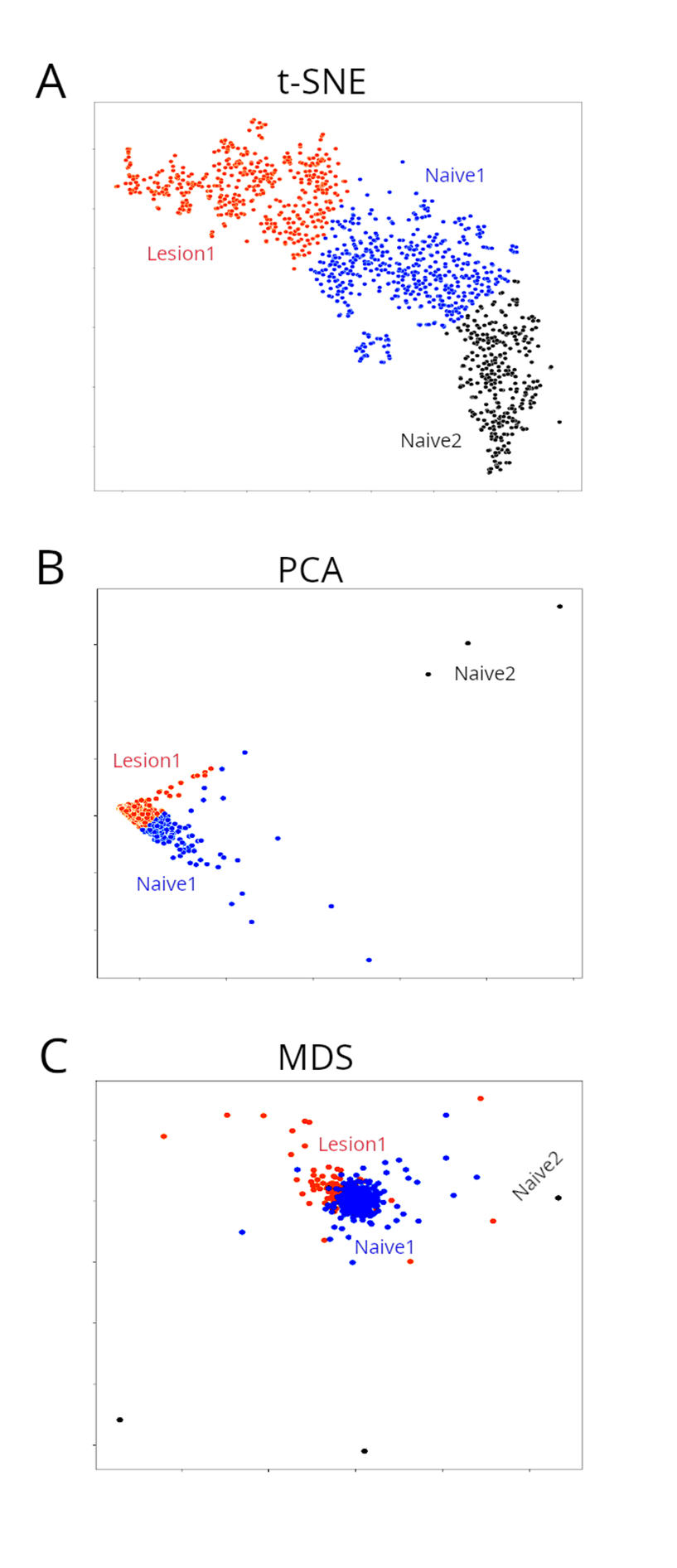

Supplement: Supplementary Figure 4 — tSNE (A), PCA (B), and MDS (C) when the microglia single cell transcriptome data was normalized using a regularized logarithmic transformation. [file Image_4.TIFF]
